# Child Whipping

**Published:** 1889-12

**Authors:** 


### CHILD WHIPPING.

"Spare the rod and spoil the child" was intended, in my estimation, in a purely metaphorical sense.

It is pretty generally conceded that cuffing children on the head or ears is frequently fraught with the most serious results—many cases of deafness and brain diseases having arisen from this practice. Evils quite as grave, I am assured by a lady physician of extensive practice, result from the punishment known as "spanking." Blows given with more or less severity and greater or less frequency in the region of the spine, will, she contends, cause serious brain or spinal trouble.

Moreover, the state of the brain and nervous system have a great effect upon the disposition, and the shock which may possibly cure one fault, may, by disordering and deranging the nervous system, produce faults of a much graver and more complicated nature.

It may console some people to know that the physician referred to does regard switching as open to the same objections as spanking, and let the followers of Solomon's precepts see that they literally use the rod and not the hand, and thus do as little harm as possible. Would that all parents could be convinced of the evil of the whole practice.—*Ladies' Home Journal.*

